# A Practical Treatise on the Diseases of the Eye

**Published:** 1855-10

**Authors:** 


Art. VII.—*A Practical Treatise on the Diseases of the Eye.* By Wm. Mackenzie, M. D., Surgeon Oculist in Scotland in ordinary to Her Majesty; Lecturer on the Eye in the University of Glasgow, and one of the Surgeons to the Glasgow Eye Infirmary. To which is prefixed an *Anatomical Introduction explanatory of a Horizontal section of the Human Eye-ball.* By Thomas Wharton Jones, Professor of Opthalmic Medicine and Surgery in University College, London, and Opthalmic Surgeon to the Hospital. With one hundred and seventy-five illustrations. From the fourth revised and enlarged London edition. With *Notes and Additions.* By Addwell Hewson, M. D., one of the Surgeons to Wills Hospital for Diseases of the Eye; Lecturer on Surgery in the Philadelphia Association for Medical Instruction, etc., etc. Philadelphia: Blanchard & Lea. 1855. 8vo., pp. 1027.

---

First Dr. Mackenzie wrote a large book on the Eye and published it in Glasgow. Then Dr. J. Wharton Jones prefixed an Anatomical Introduction explanatory of a horizontal section of the Human Eye-ball to a subsequent issue, and lastly Dr. Addinell Hewson, of Philadelphia, has annotated the most recent edition, and the work has been published in superb style by Blanchard &

Lea. It is a massive volume and its size has been increased about ten per cent. by the labors of the London and Philadelphia editors. It may be safely said to take the first rank among all publications on the diseases of which it treats. LAWRENCE on the Eye was the great book on that subject until MACKENZIE revised and enlarged his work which must now be regarded as *the* work on opthalmology.

A good deal has been said about the prevailing custom among the Philadelphia faculty of annotating English republications, and we think they have been very justly censured for so doing. Yet it occurs to us Dr. HEWSON is no more to blame than many older men who set him the example, and yet we do not remember that any of them were called to account for their misdeeds in this respect. Dr. HEWSON'S additions to the work before us are quite as respectable as J. WHARTON JONES'S are. They are a good deal more so in our opinion than Dr. KEATING'S were to the last issue of RAMSBOTHAM'S Process of Parturition, and, indeed, are quite equal to the labors of most of the editors whose names figure on the backs of so many of the works of our transatlantic brethren.

The sentiment of the profession is, however, opposed to the custom referred to, and Dr. HEWSON has happened to appear at a time when this sentiment was beginning to be expressed, and has come in for rather a large share of censure.

The editor's additions to MACKENZIE are brief, but many of them are of practical value. The first consists of a short account of the Opthalmoscope, and as we have been frequently asked and written to concerning this instrument and its uses, we copy entire the description of it given by Dr. HEWSON:

"To Prof. Helmholz, of Kœnigsberg, is due the credit of first devising an instrument, by which a sufficient amount of light can be thrown into the eye to enable us to see clearly its inner structures. His *eye speculum* consists, essentially, of a tube, with one end bevelled to an angle of 50° to its axis, on which is fastened the reflector of four parallel and highly polished slips of glass; the other end is cut square to the axis, and has adapted to it an eyepiece, containing a biconcave lens and diaphragm.

"An accident suggested this invention to Helmholtz. His friend Von Erlach, who wore spectacles, observed one day, whilst conversing with an acquaintance, that the eye of the latter became illuminated when the rays of the light from a neighboring window were reflected by his glasses into this person's eye—hence the probable reason of Helmholz using plate glass as the reflector in his ophthalmoscope.

"Since Helmholz's instrument was first made known in 1851, Coccius Meyerstein, Follin Epkens, and many others, have invented instruments but little different from it in principle. In some a plain mirror, with a hole in it, has been substituted for the reflector of plate glass; in others, a biconvex lens has been added to condense the light as it falls on the reflector; in others, the same means has been employed when either the eye of the observer or that of the patient was short-sighted, to converge the rays as they enter or pass from the latter.

"The simplest form of eye speculum yet proposed, is that described and claimed as original by Anagnostakis. Although, on the authority of an informant of Mr. Dixon, we learn that Prof. Graeff showed this very instrument, or one precisely like it, to Dr. Anagnostakis, when he was in Berlin, in 1853, some time before the publication of his paper on this subject. It consists of a small concave mirror, about 2 inches in diameter, with a focal distance of four and a half inches. In the center of this mirror there is a small hole, of a quarter of an inch in diameter. This mirror, mounted on a handle, and protected on its plated surface by a sheet of blackened copper, constitutes the apparatus described by Anagnostakis, but except in persons who are short sighted, a distinct view of the deep seated structures of the eye cannot be obtained by it, without the aid of a biconvex lens of two and a half inch focus.

"Ruete's Ophthalmoscope consists of a mirror analogous to the one just described, but of ten inch focus, and a biconvex lens, placed before the patient's eye, to increase the convergence of the rays reflected from the mirror.

"Mr. Dixon has proposed to fix the mirror into a spectacle frame, to be worn by the observer, so that both his hands may be free, and enabled, without assistance, to command the movements of the patient's lids with one hand, whilst with the other he can use the magnifying glass. He also suggests the use of mirrors of a focus corresponding to the distance of distinct vision of the observer.

"We have, on these suggestions of Mr. Dixon, made for ourselves an *extempore* instrument, at a very trifling cost, by plating with tin-foil and mercury one side of a common bi-convex lens, having selected for the purpose a lens of such convexity as would, when plated, produce a mirror of a focus to suit our own eye. This mirror we covered with dark paper through which and the plating we made a hole of about the fifth of an inch in diameter, and then mounted the mirror in one side of a common iron spectacle frame, having placed in the other a plane glass, so that the instrument would balance itself on the nose.

"The original instrument of Helmholz has not proved as efficient a one as is desirable in examining the retina; its illumination is not sufficient to give a clear view of the parts it is intended to expose. This objection cannot, however, be urged against some of the improved forms of the instrument, particularly those of Jäger and Meyerstein; but these, in common with many others, are so complicated in their construction, that we give a decided preference for the simple mirror and lens over all of them.

"*The mode of using the Instrument.*—All examinations with the eye speculum require to be conducted in a dark room, and it is also necessary that the pupil of the eye to be examined should be well dilated by the solution of atropia. A steady-burning, broad-flamed lamp will be found to be the best means of illuminating the reflector. Some recommend that the light should be placed to one side of the patient's face, and on the same plane with his and the observer's eye. This may undoubtedly be the best position for it in some instruments of a fixed angle of reflection, and may be even essential for their proper adjustment, but is not necessary where the simple concave mirror is employed. In using this we would decidedly prefer the lamp placed behind the patient's head, and so elevated that its rays will just clear his forehead to reach the observer's eye; for no matter where the light may be, we must, to throw the reflected rays into the patient's eye, compensate for the obliquity of the rays incident on the mirror, by giving a corresponding obliquity to the position of this reflecting surface. Hence, when the light is in the position we advocate, by elevating ourselves above the horizontal plane of the patient's eye, we will approach nearer the horizontal plane of the lamp, and thus diminish the angle of the incident rays, and this position will require much less obliquity of the reflector to illuminate the eye to be observed than is necessary where the light is at the side.

"The next step after arranging the position of the patient and the source of illumination, is to throw the reflection on the eye to be explored. This is done by turning the reflecting surface to the light, and then gradually changing its position until its reflection appears on the eye to be observed.

"The observer is now to apply his eye close to the orifice of the speculum (if he has not been looking through it before), and watching carefully the reflection, he is to move his head with the instrument back or forward, until the reflecting light appears about the size of the reflecting surface. The patient is then to be directed to keep his eye slightly inverted, perfectly steady, and wide open; its interior will then present a reddish hue, but none of the deep-seated structures will probably be recognizable. It is evident, then, that the proper focal adjustment has not yet been obtained. By slightly moving the head and instrument, or by interposing a biconvex lens, this will be rectified. We prefer introducing the lens here, as it has afterwards to be used. If this does not make apparent the deep-seated structures, the head and reflector are not yet in their proper position, and must be moved nearer to, or further from, the patient's eye, until the observer can distinctly detect red vessels on the yellowish red ground of the retina and choroid. If the patient's eye is inverted, as we said it should be when the lens and speculum are properly adjusted, the light will, in all probability, fall on a brilliant white circle which is the entrance of the optic nerve, and from tho center of this circle there will be readily seen two larger vessels, an artery and vein, passing up over the surface of the retina, and a similar set passing downwards over the same structure. This white circle has a diameter of one-fourth of an inch, and the contrast between it and the bright choroid shining through the retina surrounding it, is very striking. It is important that the whereabouts of this entrance of the optic nerve should be sought for at the first, as it is a valuable landmark, and its detection affords great facility in investigating the other parts; for here the illumination is brighter, being on a white surface, and the blood vessels are larger and more readily detected. Consequently, the observer becoming familiar with their appearance, will have much less difficulty in detecting them in situations not so favorable for their observation. Should the patient's eye be too much or not at all inverted, the light will fall on the surface of the retina, and the appearance presented will be that of a yellowish-red surface marked with streaks of darker red. Those dark streaks are the larger blood vessels, and by following them in the direction in which they increase in diameter, in other words, towards their origin, the observer will readily reach this landmark of the entrance of the optic nerve. Some have spoken of the facility with which they could, with the ophthalmoscope, see the pulsation in the larger arteries of the retina. The ability to detect such a phenomenon seems to us, however, rather doubtful. We believe the only mode of distinguishing the artery from the vein is by the darker color of the latter. To follow up one of these vessels in order to reach the entrance of the optic nerve, it is much better that the observer should move the instrument, the lens, and his own eye, rather than keeping these fixed to direct the patient to change the position of the eye under observation, for the latter cannot appreciate the character or exact amount of motion required to throw the light on the point sought after, and in attempting to do so, he will not unfrequently disturb completely the whole adjustment. Sometimes it will be only necessary to change the position of the convex lens to bring distinctly into view the parts we wish to observe. We need but remind the beginner of the fact, that the lens, from its peculiar property, must be moved in the opposite direction to that in which the part is, which he desires to bring into proper focus, precisely as the microscopist has to move an object which he wishes to trace under the field of his instrument.

"The vessels coming out from the optic center were stated to consist of two pairs. One, an artery and vein, passing upwards in a more or less vertical direction; the other similar in character, but passing downwards. This is the course they generally take; they will sometimes be found to vary. It is exceedingly difficult to trace them very far, from the fact that the reddened surface over which they run makes it impossible to distinguish their minuter branches; and the iris completely intercepts the view of their final distribution.

"The bottom of a healthy eye presents, as was just observed, a yellowish-red color; this color is brighter in the immediate vicinity of the optic center than on the periphery of the retina. The tint will also be found to vary in different eyes—being lighter (more yellowish) in those who are fair than in those who are dark and florid; showing that the intensity and character of the color are influenced by the pigment layer of the choroid.

"Close to the inner side or the entrance of the optic nerve, the color will be observed to be darker at one point. This Helmholtz attributes to the shadow of the semilunar fold of the retina.

"When the patient looks directly at the eye of the observer, and thus brings the axis of the two eyes in the same line, the yellow spot ot Soemmerring will come into view; the retina here is of a grayish yellow color, entirely free from any admixture of red; and has no blood vessels on its snrface.

"As regards the value of the ophthalmoscope in the diagnosis of deep-seated disease of the eye, it might justly be supposed, from what has been said of this instrument, that it would not only be indispensable in the investigation of all such diseases, but that with it there should be no difficulty in detecting the slightest pathological change in any of these structures, and in determining the exact character of the disease present, without any regard to its subjective symptoms. A great deal more, however, has been expected of, and claimed for the instrument, than it is capable of accomplishing, in the present state of its construction.

"In the first place, the great concentration of light which it produces in the eye, renders its employment highly injurious, even for a few moments of time, in the incipient stages of disease. In cases where it can be endured, its employment for any length of time sufficient to detect all the changes which have taken place, produces an excited and unnatural condition of the structures which are the subject of investigation, and might readily lead the observer astray in his diagnosis.

"In cases of more confirmed disease, it might not give rise to such annoyances and serious consequesces; but would the investigation be of any value in such cases? Are they not generally incurable? Granting such to be the case, here will be found the great value of the instrument; for it, and it alone, will often enable us to set aside, as Mr. Dixon justly observes, 'as positively hopeless, a large number of cases formerly termed *amaurotic*, or *nervous*, which were assumed to be still curable, because their real nature could not be demonstrated.'

"'We now know,' he says, 'that total disintegration of the vitreous body, detachment of the retina from its connection with the choroid, and other equally hopeless conditions of structures essential to vision, may exist without any alterations being produced in the outward appearance of the eye. In enabling us, therefore, to appreciate these conditions, the ophthalmoscope has proved of immense value.'"

